# Central and peripheral analgesia in irradiated rats: reserpine-induced pain model

**DOI:** 10.1038/s41598-026-44637-8

**Published:** 2026-04-11

**Authors:** Mostafa Saif-Elnasr

**Affiliations:** Health Radiation Research Department, National Center for Radiation Research and Technology, Egyptian Atomic Energy Authority, Cairo, Egypt

**Keywords:** Gamma-irradiation, Analgesia, Oxidative stress, Inflammation, Pain, Reserpine, Drug discovery, Medical research, Neuroscience

## Abstract

Over one-third of the world’s population suffers from persistent or recurrent pain. Thus, this study was designed to investigate the possible analgesic effect offered by a low gamma-irradiation dose, as well as to elucidate its mechanisms of action; either through central, peripheral or indirect analgesia via the attenuation of oxidative stress and/or inflammation that are the proposed components of pain induction in the reserpine-induced model. Thirty male rats were divided into: group I (Normal), group II (Reserpine), group III (Reserpine + Radiation), groups IV (Reserpine + Naloxone + Radiation), and group V (Reserpine + Diclofenac). Pain assessment by tail-flick test and Haffner’s tail clip method were performed, in addition to estimation of serum substance-P (SP) and tumor necrosis factor-α (TNF-α) levels, and brain contents of nitric oxide (NO), malondialdehyde (MDA) and dopamine (DA). Results displayed that administration of reserpine induced a significant reduction in both peripheral and central pain thresholds. However, low dose radiation induced a significant delay in pain latency in both tail-flick and Haffner’s tail clip tests, as compared to reserpine untreated group. Low dose radiation peripheral analgesic effect was similar to the analgesia offered by diclofenac. Low dose irradiation-induced analgesia revealed in Haffner’s clip test was significantly blocked upon the administration of naloxone prior to irradiation as compared to the reserpine + radiation group. Also, this study showed that the reserpine intoxication induced an elevation in rat brain MDA and NO, and serum TNF-α and SP levels, as well as a significant reduction in brain DA content as compared to the normal group. On the other hand, irradiation of reserpinized rats resulted in a restoration of all these parameters. Finally, it could be concluded that low radiation dose exerts analgesic effects through functional modulation of nociceptive pathways, potentially involving opioid-related mechanisms, as well as antioxidant and/or anti-inflammatory effects.

**Supplementary Information:**

The online version contains supplementary material available at 10.1038/s41598-026-44637-8.

## Introduction

Over one-third of the world’s population suffers from persistent or recurrent pain, costing billions per year for either health care or compensation^1^. Chronic pain is associated with conditions such as arthritis, migraine headaches, back injury, diabetic neuropathy, tempo-mandibular joint syndrome, herpes zoster, and cancer. Several of the currently available therapies of pain are either insufficient or cause uncomfortable to deleterious side effects. Untreated pain may become self-perpetuating as pain has immunosuppressive effects that make patients susceptible to subsequent diseases. It is now clear that it will be possible for patients to regain normal function, if we can effectively treat the pain despite the underlying cause^2^.

Reserpine, a rauwolfia indole alkaloid, has been broadly used as an antihypertensive agent, but major depression and musculoskeletal pain are associated with its chronic administration^3^. Diclofenac is a non-steroidal anti-inflammatory drug (NSAD) used in inflammatory and painful rheumatic and certain non-rheumatic conditions. Diclofenac is available in a number of forms of administration that can be given intramuscularly, rectally, or orally^4^. Diclofenac is an inhibitor of cyclooxygenase, and its potency is markedly greater than that of naproxen, indomethacin, and many other agents^5^.

In a different context, high irradiation doses have been previously reported to mediate central analgesia^6^. However, low radiation doses have been stated to act as an antioxidant and anti-inflammatory in many animal models. On the other hand, low irradiation doses are clinically approved for pain management in patients^7,8^ with almost no reported side effects, unlike the different classes of NSADs. NSADs serious chronic side effects require continuous patients monitoring and/or modulation of their doses/dosing regimens aiming to avoid health complications and to reduce the economic costs of pain management during the therapeutic protocol. Thus, the current study was designed to examine the possible analgesic effects offered by a low gamma-irradiation dose, as well as to elucidate its mechanisms of action; either through central, peripheral or indirect analgesia via the attenuation of oxidative stress and/or inflammation that are the proposed components of pain induction in the reserpine-induced model.

## Materials and methods

### Chemicals and reagents

Reserpine was obtained from Sigma-Aldrich Chemical Co. (St. Louis, MO, USA). Also, all reagents, kits and chemicals used in the study were purchased from Sigma-Aldrich Chemical Co. (St. Louis, MO, USA); unless otherwise specified within the text.

### Animals

Thirty male Spargue-Dawely albino rats, 110–130 g weight, were used in the current study. They were obtained from the animal house of the National Center for Radiation Research and Technology (NCRRT), Egyptian Atomic Energy Authority, Cairo, Egypt. They were supplied with standard rodent diet as well as water *ad libitum*. They were acclimatized for seven days before starting the experiment. The rats were kept at constant humidity (50 ± 5%) and temperature (22 ± 2 °C), and at 12 h light/dark cycle. All methods were reported in accordance with ARRIVE guidelines, and the study protocol was approved by NCRRT Research Ethics Committee (number: F/64A/23). All methods were performed in accordance with the relevant guidelines and regulations.

### Irradiation processing

Canadian Gamma Cell-40, (^137^Cs) irradiation unit at NCRRT was used to irradiate total-body of the rats. The rats were gamma-irradiated with 0.5 Gy as a single dose, with 0.363 Gy/min dose rate.

### Experimental design

Experimental model of pain was induced by the reserpine administration (1 mg/kg/day, s.c.) for three consecutive days^9^. The rats were divided randomly into five groups with 6 rats in each, as follows:

Group I (Normal): negative control rats received 1 ml/kg vehicle (0.5% acetic acid) subcutaneously for three days.

Group II (Reserpine): animals received reserpine (1 mg/kg/day; s.c.) for three consecutive days (i.e. days 1, 2 and 3), then pain assessment was performed on day 4. Reserpine was dissolved in glacial acetic acid, diluted with distilled water to a final concentration of 0.5% acetic acid^10^.

Group III (Reserpine + Radiation): consisted of reserpinized rats (as in Group II); gamma-irradiation at a dose level of 0.5 Gy was carried out on day 4 and pain assessment was performed 1 h after irradiation.

Groups IV (Reserpine + Naloxone + Radiation): consisted of reserpinized irradiated rats (as in Group III). On day 4, naloxone; a specific blocker of opioid µ-receptors, were injected in a dose of 8 mg/kg in saline, i.p^6^, 30 min before irradiation, and pain assessment was performed 1 h after irradiation.

Group V (Reserpine + Diclofenac): consisted of reserpinized rats (as in Group II). On 4th day, 24 h following the last dose of reserpine, rats were treated with diclofenac in a dose of 10 mg/kg in saline, i.p^11^, and pain assessment was performed 1 h after diclofenac injection.

After pain assessment, rats were sacrificed by decapitation under deep anesthesia with thiopental (50 mg/kg i.p), blood was collected in non-heparinized tubes for serum separation and further estimation of tumor necrosis factor-α (TNF-α) and substance-P (SP) levels. In addition, rat brains were isolated then stored at -80 °C for the further biochemical assessments of total nitrate/nitrite (nitric oxide (NO)), malondialdehyde (MDA) and dopamine (DA) brain contents.

### Pain assessment

Pain was assessed in animals using two methods; tail-flick test for the assessment of peripheral analgesia and Haffner’s tail clip method to assess central analgesia.

#### Tail-flick test

Analgesic activity of each of the low dose radiation (with or without naloxone) and diclofenac was evaluated by the tail-flick method^12^. Briefly, about 5 cm from the distal end of the tail of each rat was immersed in warm water maintained at 50 °C. The reaction time (in seconds) was the time taken by the rat to flick its tail due to pain. To prevent any tail tissue injury, a maximum reaction time was fixed at 15 s. Readings were recorded for each rat one hour after the drug administration or the irradiation process.

#### Haffner’s tail clip method

Pain was induced by applying mechanical pressure on the rat tail by an artery clip, the tips of which was covered with rubber tubing to avoid damage^13^. Around 1 cm away from the base of tail, the clip was applied. Rats responded to this noxious stimulus by biting the clip or the tail near the clip location. The time between the clip application and their response has been measured by a stopwatch. A cut off time was kept at 15 s^14^. Readings were recorded for each rat one hour after the drug administration or the irradiation process.

### Biochemical assessments

Brain MDA content was measured biochemically according to Mihara and Uchiyama^15^ method. Also, the brain NO was measured according to the method described by Miranda et al.^16^. In addition, serum TNF-α level was determined by enzyme-linked immunosorbent assay (ELISA) technique using a kit obtained from Koma Biotech Co. (Korea) according to the instructions of manufacturer. In addition, total brain tissue protein was quantified according to Lowry et al.^17^ method, while the serum SP level was assessed using ELISA kit (Sunlong^®^ - China). Moreover, estimation of brain content of DA was carried on according to Guo et al.^18^ method by spectrophotometric assay.

### Statistical analysis

In the current study, Graphpad^®^ Prism software was used. Data were analyzed by one-way analysis of variance (ANOVA) followed by Tukey’s post hoc test. The results are expressed as mean ± standard error (SE) of the mean. The significance level was set at p value ˂ 0.05.

## Results

### Effects of reserpine, low dose radiation with/without naloxone and diclofenac on pain latency

In this study, pain was experimentally induced in albino rats via reserpine injection. Rats were either treated with low irradiation dose (0.5 Gy) with/without pre-treatment with naloxone (central opioid blocker) or treated with diclofenac as a reference analgesic agent; for the assessment of the possible analgesia offered by low irradiation dose. Results presented in Table 1 demonstrated that the administration of reserpine induced a significant reduction in both peripheral and central pain thresholds. However, low dose radiation induced a significant delay in pain latency in both tail-flick and Haffner’s clip tests, as compared to reserpine untreated group. Unlike its central analgesic effect, its peripheral one was similar to the analgesia offered by diclofenac. On the other hand, low dose irradiation-induced analgesia revealed in Haffner’s clip test was significantly blocked upon the administration of naloxone prior to irradiation as compared the reserpine + radiation group, suggesting a central component of the analgesia offered by low radiation dose.

**Table 1:** Effects of reserpine, low dose radiation with/without naloxone and diclofenac on pain latency in male rats.

Tests	Groups				
	Normal	Reserpine	Reserpine + Radiation	Reserpine + Naloxone + Radiation	Reserpine + Diclofenac
Tail’s flick (Time in sec)	15 ± 0.89	6.22 ± 0.48^a^	10.79 ± 0.31^b^	9.81 ± 0.67^b^	11.08 ± 0.69^b^
Haffner’s clip (Time in sec)	12 ± 0.89	3.7 ± 0.27^a^	8.05 ± 0.43^b^	4.32 ± 0.32^c^	5.07 ± 0.49^b, c^

For tail’s flick: F = 24.152, *p* < 0.001. Eta-squared = 0.794.

For Haffner’s clip: F = 42.518, *p* < 0.001. Eta-squared = 0.872.

*n* = 6 rats/group.

a: denotes significance vs. normal group, b: denotes significance vs. reserpine group, c: denotes significance vs. reserpine + radiation group; at *p* < 0.05.

### Effects of reserpine, low dose radiation with/without naloxone and diclofenac on some biochemical parameters in brain (MDA, DA, NO) and serum (TNF-α, SP)

**Fig. 1:**
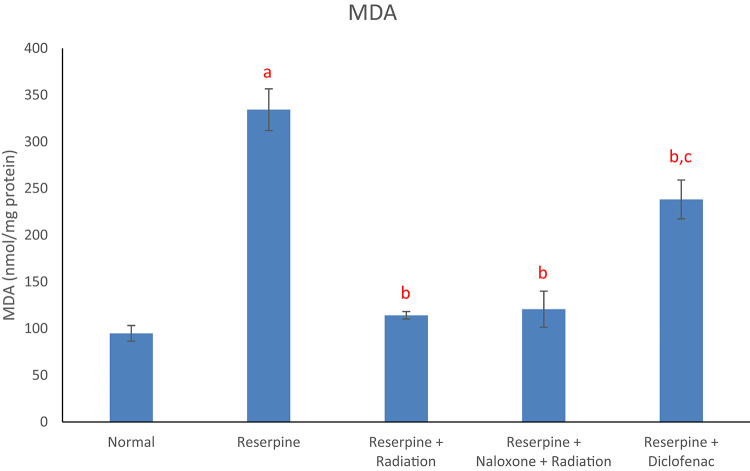
Effects of reserpine, low dose radiation with/without naloxone and diclofenac on brain MDA. F = 37.963, *p* < 0.001. Eta-squared = 0.859. *n* = 6 rats/group, malondialdehyde (MDA), a: denotes significance vs. normal group, b: denotes significance vs. reserpine group, c: denotes significance vs. reserpine + radiation group; at *p* < 0.05.

**Fig. 2:**
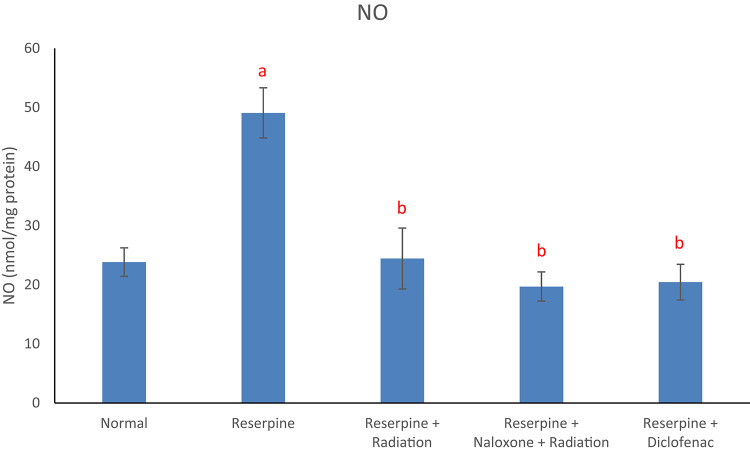
Effects of reserpine, low dose radiation with/without naloxone and diclofenac on brain NO. F = 11.377, *p* < 0.001. Eta-squared = 0.645. *n* = 6 rats/group, nitric oxide (NO), a: denotes significance vs. normal group, b: denotes significance vs. reserpine group; at *p* < 0.05.

**Fig. 3:**
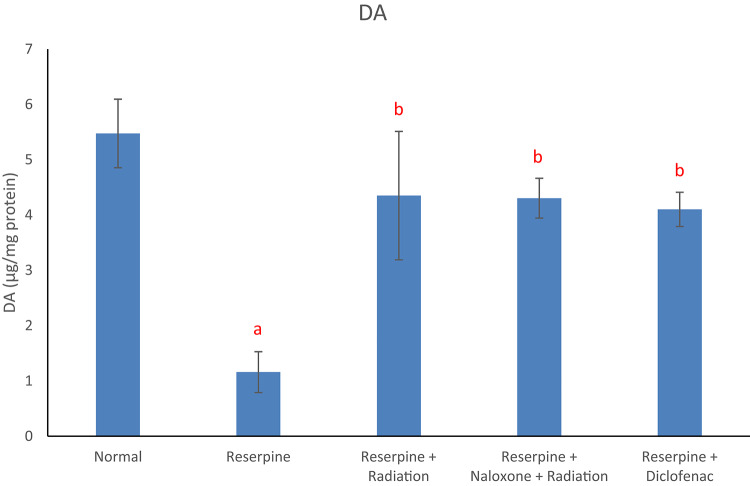
Effects of reserpine, low dose radiation with/without naloxone and diclofenac on brain DA. F = 6.212, *p* < 0.01. Eta-squared = 0.498. *n* = 6 rats/group, dopamine (DA), a: denotes significance vs. normal group, b: denotes significance vs. reserpine group; at *p* < 0.05.

**Fig. 4:**
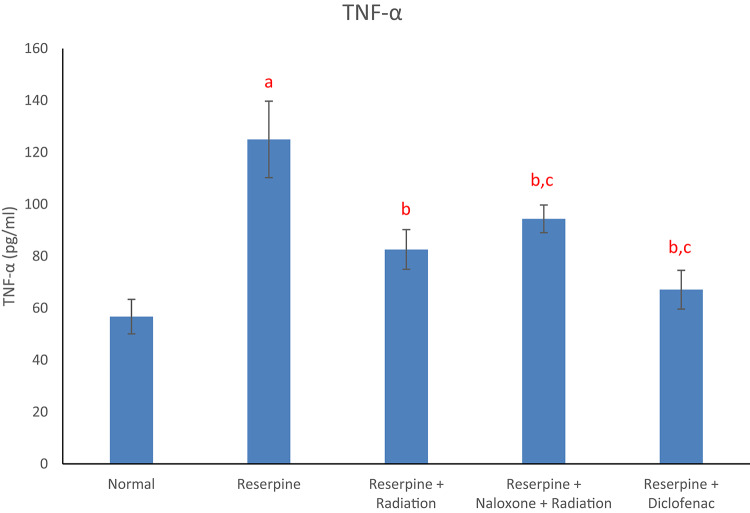
Effects of reserpine, low dose radiation with/without naloxone and diclofenac on serum TNF-α. F = 8.713, *p* < 0.001. Eta-squared = 0.582. *n* = 6 rats/group, tumor necrosis factor-α (TNF-α), a: denotes significance vs. normal group, b: denotes significance vs. reserpine group, c: denotes significance vs. reserpine + radiation group; at *p* < 0.05.

**Fig. 5:**
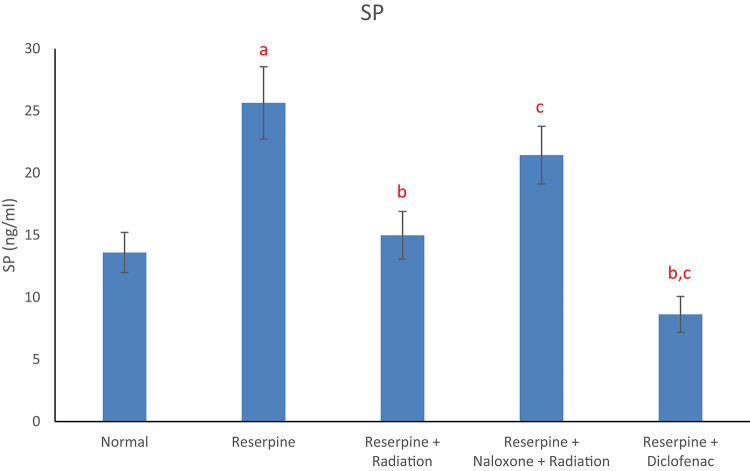
Effects of reserpine, low dose radiation with/without naloxone and diclofenac on serum SP. F = 10.122, *p* < 0.001. Eta-squared = 0.618. *n* = 6 rats/group, substance-P (SP), a: denotes significance vs. normal group, b: denotes significance vs. reserpine group, c: denotes significance vs. reserpine + radiation group; at *p* < 0.05.

Results of the current study displayed that the reserpine intoxication induced an elevation in rat brain redox biomarkers (MDA and NO) as compared with the normal group (Figs. 1 and 2). Reserpine group also showed a significant decrement in brain DA content (Fig. 3) as well as a significant rise in serum level of each of TNF-α and SP (Figs. 4 and 5) following reserpine administration as compared to the normal group. On the other hand, irradiation of reserpinized rats resulted in a reduction in brain MDA and NO contents (Figs. 1 and 2) associated with a restoration in brain DA content (Fig. 3) as compared to the reserpine group. Moreover, irradiation at 0.5 Gy attenuated the reserpine-induced rise in serum TNF-α and SP levels when compared to reserpine group (Figs. 4 and 5). In another context, administration of naloxone prior to irradiation showed no significant changes in any of the biochemical parameters assessed; except the serum TNF-α and SP levels as compared to reserpine + radiation group. As expected, diclofenac was able to attenuate both the oxidative stress and inflammatory status and the reserpine-induced pain, as compared to the reserpinized untreated group.

## Discussion

Exposure to ionizing radiations such as gamma-rays has been suggested to have a hormetic effect at doses not higher than 0.5 Gy^19,20^. This hormetic effect has been suggested to be the result of reduction in oxidative stress^21^ with a possible consequent anti-inflammatory effect^22,23^.

Low radiation dose was found to have pain improvement effect^24,25^. In the late 1970s, following the discovery of endogenous opioids, the ionizing radiation effects on the mechanism of opioid were studied; when Teitelbaum et al.^26^ observed that radiation exposure led to behavioral and biochemical responses that look like those observed after the exogenous opiate, morphine administration. In the current study, the analgesic effects of low dose gamma-irradiation have been studied in a reserpine-induced pain model; with or without the administration of the opioid receptor blocker naloxone, using diclofenac as a reference analgesic agent. Pain latency has been found to decrease significantly following the administration of reserpine as indicated by a significant decrease in both peripheral (tail-flick test) and central (Haffner’s tail clip test) pain thresholds, an effect that was significantly attenuated in rats exposed to radiation. On the same line, the levels of beta-endorphin were reported to be raised in irradiated mice^27,28^. Moreover, Teskey and Kavaliers^29^ displayed that, CF-1 mice exposure to ionizing radiation doses as low as 2.5 Gy caused an elevation in their nociceptive thresholds in the hot-plate test, indicating radiation-induced analgesia. On the other hand, the delayed pain responses observed following radiation were blocked and reversed by the administration of naloxone, suggesting a central component of the analgesia offered by low radiation dose. Similar findings were reported in the study carried by Castellano and Oliveiro^30^; in which the mechanism of radiation-induced analgesia has been proposed to be either mediated by overall radiation effects on radiosensitive tissues in the body, or by its direct effect on the nervous tissue in central nervous system (CNS) such as the opioid receptors^6^. Another supporting observation was proven in Miyachi’s^31^ study who also reported analgesic effects of radiation by repeated mice irradiation with a daily dose as low as 0.5 Gy for six successive days.

SP belongs to the tachykinin neuropeptide family. It is an undecapeptide (i.e., 11-amino acid long peptide) which is encoded by the pre-protachykinin-A (TAC1) gene^32^. SP is synthesized and secreted by nerve cells. Dendritic cells, macrophages, monocytes, eosinophils, mast cells, and lymphocytes also synthesize SP^33^. Several studies have stated a widespread SP distribution in the mammalian peripheral and central nervous systems^34–36^. Several studies have also stated a widespread of its receptor distribution in the mammalian CNS in addition to the peripheral tissues^37–39^. Furthermore, SP exists in body fluids including the cerebrospinal fluid and blood. It mediates the crosstalk between the immune and nervous systems and regulates the functions of the cell by diverse mechanisms (endocrine, paracrine, autocrine, and neuroendocrine)^40^. SP is widespread through the human body. Additionally, it has an important role in several pathophysiologic and physiologic functions as pain, inflammation, and angiogenesis^41^.

SP is an active neuropeptide in the CNS and there are studies which show the role of SP, as it lower pain thresholds^42^. In the existing study, we found increased serum level of SP following reserpine administration to rats, and this finding is in concurrence with Arora et al.,^10^ who demonstrated that reserpine administration displayed a significant elevation in the levels of SP in both hippocampus and cortex regions of the rat brain. On the other hand, we found that irradiation at 0.5 Gy attenuated the reserpine-induced rise in serum SP level.

Our results demonstrated a significant reduction in brain DA content following reserpine administration. This agreed with the previous studies which revealed that reserpine markedly decreased DA, norepinephrine (NE), 5-hydroxytryptamine (5-HT) contents in rat brain^43,44^. Reserpine, a known vesicular monoamine transporter 2 inhibitor, interferes with the monoamines reuptake and storage in vesicles, by irreversibly binding to the vesicle monoamine transporter it depletes biogenic amines from the nervous system to attenuate biogenic amine-mediated control^45,46^. DA has been implicated in modulation of pain within the CNS, with DA receptor agonists demonstrating efficacy in decreasing experimental allodynia and hyperalgesia^47^. serotonin and NE play essential roles in the pathways of descending analgesia from the midbrain periaqueductal gray matter to the spinal cord^48^. In patients with fibromyalgia, changes in these neurotransmitters can contribute to exacerbation of central pain symptoms and noxious discomfort^49^. The sustained monoamines decrease supports the hypothesis that disruptions in monoaminergic regulation are implicated in the nociceptive pain development, particularly in the CNS and skeletal muscle^50^. Results of Shibrya et al.,^43^ showed that, low dose of gamma irradiation (0.5 Gy) antagonized DA, NE and 5-HT contents depletion induced by reserpine in brain of rats which fall in the same line with our results where irradiation of reserpinized rats resulted in a restoration in brain DA content. Also, Mostafa et al.,^51^ stated that treatment with low dose gamma-radiation alleviated bee venom suppression of brain mediators (serotonin, NE, and DA). Similarly, Liang et al.^52^ cleared that a low mice whole-body gamma-irradiation dose caused neuroprotection against striatal dopaminergic nerve fibres damage mediated by l-methyl-4-phenyl-1,2,3,6-tetra-hydropyridine (MPTP). This low whole-body irradiation dose neuroprotective effect might be due to monoamine oxidase activity inhibition in the hippocampus, cerebellum and cerebral cortex. The observed elevation in the content of DA in irradiated rats in the absence of reserpine in Shibrya et al.,^43^ study further supported this mechanism.

Kang et al.^53^ illustrated that a low irradiation dose could rescue or ameliorate individuals at risk from several types of neuronal damages, and displayed a protective effect in most mammalian cells. Moreover, Kataoka et al.^54^ stated that it has several beneficial effects in many disorders. Various studies have illustrated the useful low dose gamma-radiation adaptive response, which might be effective in preventing many reactive oxygen species (ROS)-related diseases in brain. It has been stated that gamma-irradiation of whole-body at a dose of 0.5 Gy activated antioxidant function and suppressed brain injury induced by cold in mice^55^. This concurred with the Kojima et al.^56^ findings who demonstrated that gamma-irradiation (0.5 Gy) of whole-body ameliorated brain antioxidant defense induced by MPTP injection. Abdel-Rafei et al.^57^ found similar results where gamma-irradiation at a dose of 0.5 Gy afforded neuroprotection against hepatic encephalopathy induced by thioacetamide. Interestingly, the low irradiation dose effects on antioxidant defense system and glutathione (GSH) have been illustrated in different rodents’ organs. It was postulated that the beneficial low irradiation dose biological effects caused by an amelioration in antioxidant enzyme activities and GSH contents in the liver, spleen, brain, lungs, macrophages, thymus, natural killer cells and bone marrow^58^.

Another proposed mechanism; introduced herein, for radiation-induced analgesia was the attenuation of free radicals’ production which implicates both decrement in the oxidative stress and the inflammatory component of pain production cascade. In the current study, low irradiation dose resulted in a significant attenuation in reserpine-induced oxidative stress and inflammatory response. An observation that is consistent with those stated in Yamaoka et al.^59^, Kojima et al.^56^ and Large et al.,^22^ studies. The brain is more susceptible to oxidative stress than other organs or systems due to its high content of unsaturated membrane lipids, autoxidizable neurotransmitters, high oxygen utilization, and low antioxidant defense molecules levels^60^. In the current experiment, reserpine intoxication induced an increment in rat brain redox biomarkers (MDA and NO). Shibrya et al.,^43^ indicated that administration of reserpine enhanced brain tissue lipid peroxidation, as indicated by significant elevation in content of MDA, parallel to decreased brain content of GSH. Moreover, content of NO was also significantly increased in reserpinized rats brain tissue (Shibrya et al., 43), suggesting that NO is an important messenger molecule in signal transduction pathways that enhance nociceptive transmission in the CNS^61^. Also, the study of Khodir et al.,^44^ demonstrated that reserpinized rats displayed significantly higher brain MDA levels, and lower brain activities of superoxide dismutase (SOD), compared to the normal rats. Reserpine, a known monoamines depleter, can induce oxidative stress by DA autoxidation and oxidative catabolism stimulation via monoamine oxidase^62^. These processes can cause quinones and hydrogen peroxide production in DA neurons, disrupting their neuronal terminals redox status, as demonstrated by decreased SOD and GSH levels^63^. Many studies have suggested that NO is a toxic molecule mediating the DA cells death. It was shown that the NO concentrations in the prefrontal cortex and striatum elevated in response to the oxidative stress induced by reserpine^64^. Within the CNS, NO is an important physiological signaling molecule involved in the vascular tone control and neurotransmission, but neurotoxicity might occur when produced in excess^65^.

Results of the existing study displayed that irradiation of reserpinized rats resulted in a reduction in brain contents of MDA and NO, which are in agreement with Shibrya et al.,^43^ who indicated that low gamma-irradiation dose (0.5 Gy), attenuated the increase in contents of MDA and NO induced by reserpine as well as the decrease in content of GSH in brain tissues. These results also agreed with preceding studies which demonstrated that gamma-irradiation of total body with 0.5 Gy elevated the antioxidant substances as SOD, GSH and catalase in various normal mice organs, and reduced the levels of lipid peroxide^66^.

Consequently, Liang et al.^52^ suggested various mechanisms for the low irradiation dose-induced neuroprotection: the immune function activation, the increase in levels of GSH and radiation hormesis-induced enzymatic DNA repair. Shibrya et al.,^43^ showed an elevation in brain GSH due to radiation in rats exposed to gamma-radiation of whole body alone. Furthermore, Kipnis et al.^67^ stated that the low gamma-irradiation dose was accompanied by an elevated activated T cells incidence, leading to self-reactive T cells accumulation in the injured CNS and neuroprotection.

In the current study, a significant increase in serum pro-inflammatory marker level, TNF-α, was showed following reserpine administration which indicate the inflammatory effect of reserpine. This inflammatory effect may be contributed to reserpine-induced oxidative stress. As indicted previously, ROS can promote inflammation by stimulation of the transcription factor nuclear factor-kappa β, which promotes pro-inflammatory cytokines production, including TNF-α^68^ as well as activates inducible NO synthase^69^. The current study results are in concurrence with Khodir et al.,^44^ who revealed that brain inflammatory markers levels (prostaglandin E2 and TNF-α) were substantially greater however brain anti-inflammatory marker levels (interleukin-10 (IL-10)) were considerably lower in the group of reserpine than those of the normal group. Also, Arora et al.,^10^ found increased TNF-α and IL-1β levels in the cerebral cortex and hippocampus of reserpinized rats. On the other hand, we found that irradiation at 0.5 Gy attenuated the reserpine-induced increase in serum TNF-α levels. Similarly, Mostafa et al.,^51^ displayed that treatment with low gamma-radiation dose (0.5 Gy) after bee venom administration tended to normalize brain pro-inflammatory cytokines IL-6, TNF-α and IL-1β levels. On the same line, previous studies demonstrated the anti-inflammatory effects of low radiation dose^22,23^.

In the present study, whole body exposure to low gamma-irradiation dose employed as an experimental model to investigate systemic and mechanistic effects of low radiation dose on pain relief process, although clinically low-dose radiotherapy for pain relief is typically applied in a localized manner to the affected tissues. Therefore, the current analgesic and biochemical effects observed in our experimental model provide insights that may be relevant to clinical localized radiotherapy, where similar pathways may contribute in pain relief, and allow the assessment of systemic responses and provide mechanistic support for the analgesic effects of low dose radiation therapy. Additional future studies employing localized irradiation protocols are needed to validate the clinical applicability of the present findings.

## Conclusion

Finally, it could be concluded that low gamma-radiation dose has analgesic effects in the reserpine-induced pain model. These effects appear to involve functional modulation of nociceptive pathways at spinal and supra-spinal levels, potentially including opioid-related mechanisms, and might be mediated through radiation-induced antioxidant and anti-inflammatory mechanisms, as evidenced by the restoration of oxidative stress and inflammatory biomarkers.

However, the limitations of this study are using a single animal model and a single radiation dose, so further studies are needed to explore these effects in other models and with varying low doses of radiation. Likewise, the used sample size is small, so further studies using larger sample size are needed. Also, the study assesses pain and biochemical parameters only at a single time point (1 h post-treatment), so conducting time course studies are needed to evaluate the duration and dynamics of the analgesic effects and biochemical changes induced by low radiation dose.

## Supplementary Information

Below is the link to the electronic supplementary material.

Supplementary Material 1

## Data Availability

The data supporting the findings of this study can be available upon reasonable request.
